# Gold Nanoparticles Coated With Hydrophobin‐ProteinA Fusion Protein: Development of a Versatile Immunosensing Platform

**DOI:** 10.1002/bit.70069

**Published:** 2025-09-13

**Authors:** Paola Cicatiello, Bartolomeo Della Ventura, Giulia Fichera, Raffaele Velotta, Paola Giardina, Alessandra Piscitelli

**Affiliations:** ^1^ Department of Chemical Sciences University of Naples Federico II Naples Italy; ^2^ Department of Physics “E. Pancini” University of Naples Federico II Naples Italy

**Keywords:** AuNPs aggregation, colorimetric biosensor, functional amyloid proteins, localized surface plasmon resonance, surface functionalization

## Abstract

Advancing immunosensing technologies hinges on the development of next‐generation surface functionalization methods, as the precise anchoring of antibodies on transducer interface is essential for achieving high sensitivity and selectivity. Among the diverse methodologies explored, bioengineered materials have shown significant potential to improve antibody orientation, stability, and functional performance. In this study, we present a chimeric protein created by fusing the adhesive Class I hydrophobin Vmh2 from *Pleurotus ostreatus*, with the Fc‐binding region of protein A from *Staphylococcus aureus* (SpA). This fusion protein spontaneously adheres on gold nanoparticles (AuNPs) without requiring chemical modification, forming a robust bio‐interactive layer for antibody attachment. The platform's adaptability and effectiveness were assessed using an immunoglobulin specific to a fungal laccase to establish the performance of the system, and antibodies against two clinically significant targets‐ mesothelin, a tumor‐associated glycoprotein, and the SARS‐CoV‐2 spike protein‐ to showcase the diagnostic potential of the system. A two‐step method based on the induced aggregation of the AuNps not bound to the analyte allows underscoring the platform's promise in biosensing applications. Overall, this approach represents a sustainable, versatile, and low‐cost route for fabricating biologically active surfaces, with wide‐ranging relevance in medical diagnostics, environmental analysis, and biotechnological innovation.

## Graphical abstract

The biosensor uses a two‐step method involving Ab‐Vmh2/SpA‐AuNPs. Gold nanoparticles are functionalized with Vmh2/SpA, antibodies and then mixed with analytes. HCl induces aggregation in unbound particles enabling detection through spectral analysis based on analyte interaction and nanoparticle aggregation behavior.

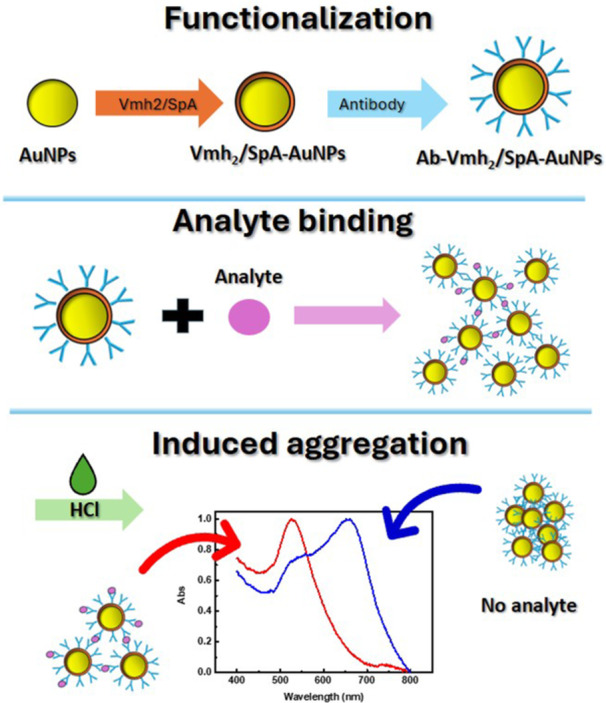

## 1 Introduction

The research for innovative surface functionalisation strategies is particularly challenging since this step plays a pivotal role for many methodologies, affecting their quantitative and qualitative aspects (Lavilla et al. 2014). Among these methodologies, immunosensing is an outstanding analytical method that utilizes the highly specific molecular recognition of antibodies (Gao et al. 2022). The continuous research and innovation in antibody immobilization strategies, signal amplification, and immunoassay concepts witnesses a need for further advances in this field to achieve rapid analysis time, low‐cost, and superior bioanalytical performance. Due to its high specificity and sensitivity, immunosensing is exploited in various fields such as medical diagnosis, food analysis, and environmental monitoring. All these applications benefit from the right orientation of the antibodies on the surfaces of a transducer, thus avoiding the false negatives and ensuring low limits of detection (LoD). To achieve the maximum recognition capacity of immunosensors and guarantee their long‐term stability, the ideal antibody immobilization has to provide two further characteristics (Gao et al. 2022; Hashemi et al. 2020), such as firmly conjugation on surfaces with the least possibility of leaching during analysis procedure (Afkhami et al. 2017), and prevention of unspecific interaction on the final support, avoiding false positives (Lin and Li 2020). A wide range of antibody immobilization strategies have been developed during the last three decades (Vashist and Luong 2018) broadening by passive adsorption procedure to cross‐linking reactions. The protein A (SpA), a cell wall protein from *Staphylococcus aureus* able to bind to Fc region of immunoglobulin G (IgG), has been highly used in biochemical, biotechnological, and medical applications, for the purification and detection of antibody, and immunological analysis (Rigi et al. 2019).

New and tangible opportunities to immobilize antibodies on chemically inert surfaces are provided by sticky self‐assembling amyloid proteins, which can integrate bio‐hybrid functional materials into immunosensors (Duan et al. 2020). Indeed, biosensors based on amyloid proteins show an enhancement of sensitivity with respect to other reported systems thanks to the achievement of a high antibody density together with their correct orientation (Men et al. 2009). Noteworthy members of the functional amyloid family are Class I hydrophobins (HPBs), small fungal proteins able to efficiently and easily adhere to several conventional and nanostructured surfaces by direct deposition, without the need of harsh chemical processing (Cicatiello et al. 2020). Namely, the class I HPB Vmh2 from *Pleurotus ostreatus* has been explored to develop a sustainable, facile, fast, cost‐effective, universal, and eco‐friendly strategy to obtain functionalised surfaces. The Vmh2 “adhesive” layer has been used for the immobilization of different active proteins (Cicatiello et al. 2018; Patel et al. 2016). Design and production of chimeric proteins, made by the genetic fusion of the Vmh2 adhesive moiety and biotechnologically relevant proteins to be immobilized on different surfaces, have expanded the potential of Vmh2 by enabling one step anchoring of active proteins onto various surfaces (Pennacchio et al. 2018; Pennacchio et al. 2022; Sorrentino et al. 2021). Among these chimerae, fusion proteins combining Vmh2 and the functionality of the single‐chain fragment variables (ScFvs) of two antibodies able to recognize marine toxins have been efficiently produced, and electrochemical and colorimetric biosensors developed (Stanzione et al. 2021).

To the aim of achieving a more versatile system, herein a new fusion protein was produced by combining the self‐assembling adhesive properties of Vmh2 and the functionality of the SpA. The recombinant chimera was produced in the yeast *Pichia pastoris*, and its functionality was verified after immobilization on gold nanoparticles (AuNPs) using an environmentally friendly method that required no chemical derivatization. Different binding tests with different antibodies were carried out with the aim of showing the versatility and efficiency of the system. The evaluation of the system was carried out using an antibody that recognizes a laccase (Giardina et al. 2010), a phenol oxidase enzyme easily detectable through a sensitive colorimetric assay. Subsequently, as a proof of concept, the effective use of the system was demonstrated using two antibodies for the detection of diagnostically relevant proteins, mesothelin and the SARS‐CoV‐2 spike protein. Mesothelin is a cell‐surface glycoprotein that is normally expressed in mesothelial cells and is considered a potential biomarker for several cancers due to its elevated expression in malignant cells. It is cleaved and released into the bloodstream as soluble mesothelin‐related protein (SMRP) at the critical concentration of about 1.9–2.5 nM (Sorino et al. 2023). On the other hand, the spike protein is a key structural protein found on the surface of the virus that causes COVID‐19, SARS‐CoV‐2, and it is the primary target in diagnostics and therapeutic development (Zhou et al. 2023).

## 2 Materials and Methods

### 2.1 Vector Construction

The synthetic gene encoding the hydrophobin Vmh2 from *P. ostreatus*, a linker (Gly4Ser)_3_, the SpA from *S. aureus* (accession number 1314205A, sequence from 27 to 325 mutating the four potential N‐glycosylation sites in Q), and a His‐tag was designed and optimized according to the *P. pastoris* codon usage. The gene product was restricted with *BsaI* and ligated into the corresponding site of the pJGG_αkR vector in‐frame with the α‐factor (signal peptide) under the control of the constitutive *GAP* promoter, yielding the recombinant plasmid pJGG_Vmh2/SpA (Life Technologies). The recombinant plasmid was linearized by *BsiW*I and transformed into *P. pastoris* BG‐23 (a protease‐deficient strain, lacking proteinase A (*pep4*) and subtisilin 2 (s*ub2*)) and BG10 (a protease‐producing strain).

### 2.2 Transformation of *P. pastoris* and Screening of the Clones

The transformation of *P. pastoris* cells was carried out following a protocol already reported (Anzevino et al. 2024a; Pennacchio et al. 2022). The colonies were collected, inoculated in Buffered Methanol Complex Medium (BMMY) (13 g/L Yeast Nitrogen Base, 10 g/L Yeast extract, 100 mM Potassium phosphate pH 6, 20 g/L Pepton, 4 × 10^−5^ g/L biotin, 5 g/L methanol), and grown at 28°C on a rotary shaker (250 rpm). Aliquots were daily withdrawn and assayed for cell density and used for the Western blot analysis.

### 2.3 Expression and Purification of the Fusion Protein Vmh2/SpA

Pre‐inoculum preparation of the recombinant selected clone was performed at 28°C overnight in BMMY and then diluted 1:100 in the same medium. The recombinant protein was assayed daily for 6 days and the cells were removed by centrifuging for 20 min at 7000 rpm at 4°C. Parameters such as growth temperature (20°C–28°C), the presence of the serine protease inhibitor phenylmethanesulfonyl fluoride (2 mM PMSF) and different carbon sources (5 and 10 g/L methanol, 5 and 10 g/L glucose, 5 and 10 g/L glycerol) were tested. The recoverd samples were subsequently analyzed by Western blot analysis to determine the best growth conditions. The optimized growth conditions were used to produce the chimera protein. Then, the supernatant was recovered, concentrated, and dialyzed towards 50 mM Tris‐ HCl buffer, pH 8.0, using Amicon system with Ultracel YM‐10 membrane (Merck, Darmstadt, Germany). The protein concentration was determined using the Bradford method (BioRad) according to the manufacturer's instructions and using bovine serum albumin (BSA) as a standard.

### 2.4 Western Blot Analysis Analysis

To assess the production the recombinant proteins, a Western blot analysis was carried out exploiting the presence of the His‐tag at the C‐terminus of the chimera. Protein samples were loaded on SDS‐PAGE (12.5%) and transferred to a PVDF membrane using an electroblotting transfer apparatus (Trans‐Blot Semi‐Dry Transfer Cell, Bio‐Rad, Segrate (MI), Italy). The protein detection was carried out using a monoclonal peroxidase–conjugated anti‐polyHistidine antibody at a 1:2500 ratio (Sigma‐Aldrich, Saint Louis, Missouri, United States). The membranes were developed by using a chemiluminescent substrate WESTAR ƞC 2.0 (Cyanagen). To relatively quantify Vmh2/SpA protein bands from Western blot film, a calibration curve with a purified reference protein carrying the same affinity tag was constructed. The areas under each band were calculated using ImageJ software and interpolated.

### 2.5 Functionalization of AuNPs

AuNPs were synthesized and characterized by SEM images as previously reported (Anzevino et al. 2024a; Pennacchio et al. 2024). Then 1 mL of AuNPs was centrifuged at 3000 g for 30 min, the supernatant discarded and the AuNP pellet resuspended in ultrapure water. A solution of 50 μg/mL Vmh2/SpA was added step‐by‐step to the colloidal solution under slight stirring, obtaining AuNPs named Vmh2/SpA‐AuNPs. The same procedure was carried out using commercial SpA (Rockland PA00‐00 Immunochemicals, Pottstown, United States) in equimolar amounts, obtaining AuNPs named SpA‐AuNPs. UV−Vis absorption spectroscopy and dynamic light scattering (DLS) were used here to characterize both bare and functionalized AuNPs and to study their optical response in the presence of the coating protein. The UV−vis absorption spectra were recorded at different times from 400 to 700 nm on a Jenway 6715 UV/vis spectrophotometer with 0.1 nm resolution and 0.2 nm spectral bandwidth. DLS measurements were conducted using a Zetasizer Nano ZS (Malvern Instruments) equipped with a 633 nm He−Ne laser and an avalanche photodiode detector placed at the detection angle of 173°. Zetasizer Nano ZS was also used to perform ζ‐potential measurements based on laser Doppler microelectrophoresis.

### 2.6 Antibody Immobilization on Functionalized AuNPs

150 µL of antibody solution in PBS buffer were added step by step to 1 mL of Vmh2/SpA‐AuNPs or SpA‐AuNPs and incubated for 1 h at 4°C by a rotary shaker. After that, the solution was centrifuged and the pellet of both Ab‐Vmh2/SpA‐AuNPs and Ab‐SpA‐AuNPs was resuspended in ultrapure water.

### 2.7 Ab‐Vmh2/SpA‐AuNPs Aggregation

The type and concentration of the aggregating agent were first optimized. Three different aggregating agents—HCl, NaCl, and PBS—were tested at varying concentrations to optimize the saline conditions for achieving the desired colorimetric response. Specifically, HCl and NaCl were tested in the 1–20 mM range, and PBS in the 3–60 mM range. After adding the aggregating agent and incubating for 1 min, absorption spectra from 400 to 750 nm were recorded and analyzed. HCl at its highest tested concentration showed the greatest peak shift and was chosen as the aggregating agent (Supporting Information S1: Figure S1). Human serum samples were collected from healthy subjects and used to assess potential interferences.

### 2.8 Biosensor Development Through the Two‐Step Method

The biosensor is based on the two‐step method that relies on the aggregation of Ab‐Vmh2/SpA‐AuNPs not bound to the analyte. The AuNPs functionalized with Vmh2/SpA were tested against three rabbit polyclonal antibodies: Anti‐laccase poxa1b antibody (Palmieri et al. 2000), Anti‐Mesothelin antibody (Abbexa, Cambridge, United Kingdom) and Anti‐SARS‐CoV‐2 S1F antibody (RayBiotech, Georgia, United States).

The whole detection protocol can be sketched in various steps:

*1) Antibody immobilization*: 150 µL of antibody solution (from 1.8 to 30 μg/mL) in PBS buffer were added slowly to the Vmh2/SpA‐AuNPs and incubated for 1 h at 4°C by rotary shaker, after that the solution was centrifuged and the pellet was resuspended in ultrapure water.

*2) Sample preparation*: serial dilution of the target analytes in water from 17 ÷ 167, 25 ÷ 125, and 13 ÷ 260 nM range for laccase, mesothelin and spike, were prepared respectively.

*3) Analyte binding*: the Ab‐Vmh2/SpA‐AuNPs were mixed with the target analytes, with a µL ratio 1:1, at different concentrations for 10 min to allow the interaction.

*4) Induced aggregation*: after the incubation, an induced aggregation of the Ab‐Vmh2/SpA‐AuNPs was provoked by adding 20 mM HCl to the mixture.

*5) UV‐Vis measurement*: after a further 1 min from the induced aggregation, the absorption spectrum from 400 to 750 nm was measured and analyzed.

### 2.9 Limit of Detection (LoD)

The LoD was calculated according to the formula: LoD = mean_control + 3 × SD_control (where SD is the Standard Deviation). The error associated with the LoD was estimated by dividing the average SD of the controls by the square root of the number of replicates (*n*), and then multiplying the result by 3.

## 3 Results and Discussions

### 3.1 Vmh2/SpA Protein Production

The fusion protein Vmh2/SpA was produced in *P. pastoris*, and recovered in the culture medium. Different strategies were tested since its production was compromised by proteolytic degradation. To minimize this, a serine protease inhibitor was added daily and the culture temperature was reduced to 20°C. While this approach reduced hydrolysis, as confirmed by the Western blot showing a single 44 kDa band, the overall protein yield remained low (Supporting Information S1: Figure S2). To improve its production, a protease‐free strain of *P. pastoris* was employed, testing various growth conditions including temperature, carbon sources, and PMSF supplementation. The carbon source type and growth temperature had minimal impact on short‐term expression. The best results were obtained at 28°C with PMSF addition, yielding the maximum of Vmh2/SpA production after only 1 day (Supporting Information S1: Figure S2). The secreted proteins were recovered from the culture medium, concentrated, and dialyzed by ultrafiltration, and directly used to functionalize AuNPs.

### 3.2 Functionalization of AuNPs With Vmh2/SpA

AuNPs were selected as the platform for biosensor development due to their unique optical properties, particularly their strong localized surface plasmon resonance (LSPR) band in the visible spectrum. The colorimetric biosensor developed in this study leverages the visible color change induced by AuNPs aggregation, which arises from interparticle plasmonic coupling and results in a red shift and broadening of the LSPR band. This well‐established phenomenon provides a straightforward and sensitive visual readout. The synthesized AuNPs exhibited an average core diameter of approximately 20 nm and displayed a characteristic plasmonic absorption peak centered at ~518 nm. This LSPR peak served as a key indicator throughout the surface functionalization process. Functionalization by Vmh2/SpA was performed in a stepwise manner to avoid undesired AuNPs aggregation. Indeed, protein solutions at appropriate concentrations were slowly added to achieve stable and reproducible functionalization without inducing colloidal instability. The concentration of Vmh2/SpA was approximately quantified by Western blot, using a calibration curve generated with a reference protein carrying the same affinity tag. This determination allowed the use of SpA as a control, in equimolar amount of Vmh2/SpA (Supporting Information S1: Figure S3).

**Figure 1:**
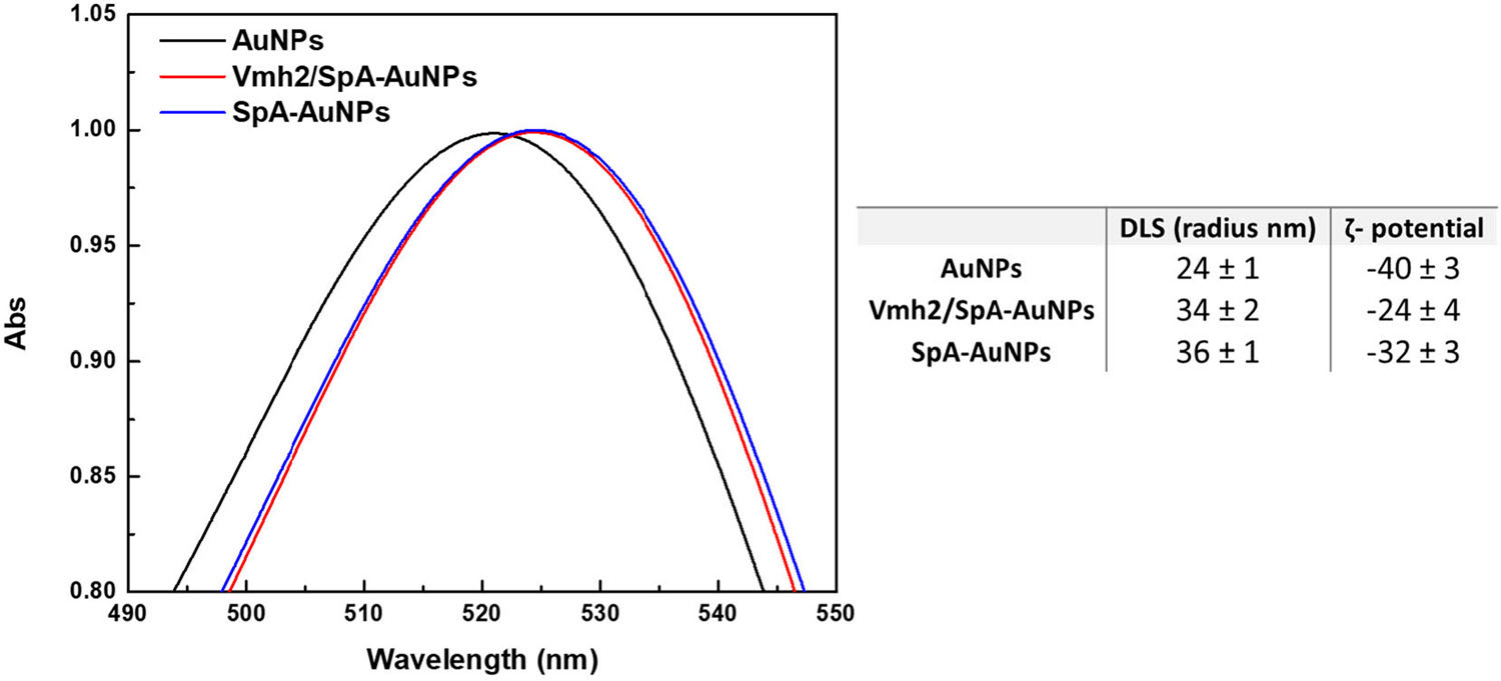
UV‐Vis analysis, DLS, and ζ‐potential of AuNPs after functionalization with Vmh2/SpA and SpA.

To monitor and confirm successful surface modification, a combination of UV–Vis spectroscopy, DLS, and ζ‐potential measurements was employed. UV–Vis analysis revealed a red shift in the LSPR peak in both cases, Vmh2/SpA‐AuNPs and SpA‐AuNPs, indicating the formation of a protein corona around the nanoparticle core. The shift of the absorbance peaks at longer wavelengths was consistent with a change in the refractive index due to surface coating (Figure 1). DLS analysis further supported the functionalization results, showing an increase in hydrodynamic diameter of the bare AuNPs upon binding of the Vmh2/SpA chimera and of SpA protein. ζ‐potential measurements showed that the native negative charge of bare AuNPs was partially shielded upon protein adsorption for both proteins. The reduction in surface charge is consistent with the successful attachment of the protein layer and reduced electrostatic repulsion among particles (Figure 1). These results demonstrate that both proteins can functionalize AuNPs.

To evaluate whether the functionalized AuNPs enable effective antibody immobilization, the anti‐laccase antibody was used, and the enzymatic activity of the target analyte served as a functional readout. Both Vmh2/SpA‐AuNPs and SpA‐AuNPs types were incubated with the anti‐laccase antibody, followed by exposure to the enzymatically active laccase analyte. Subsequent enzymatic assays revealed a clear performance difference between the two systems. A 65% higher activity was exhibited in the case of Vmh2/SpA‐AuNPs with respect to those functionalized with SpA alone. Moreover, the colloidal dispersion of Vmh2/SpA‐AuNPs was stable at 4°C for at least 4 months, while in the same conditions SpA‐AuNPs precipitated, displaying a shorter stability, as assessed by UV‐Vis spectra analysis (Figure S4).

These results strongly suggest that the chimeric protein promotes the SpA right orientation, accessibility, or density of antibody molecules on the nanoparticle surface, ultimately leading to a more efficient antigen recognition and signal generation. For these reasons, the following experiments were conducted using Vmh2/SpA‐AuNPs.

### 3.3 Evaluation of the Vmh2/SpA‐AuNPs Performance Against Different Antibodies

**Figure 2:**
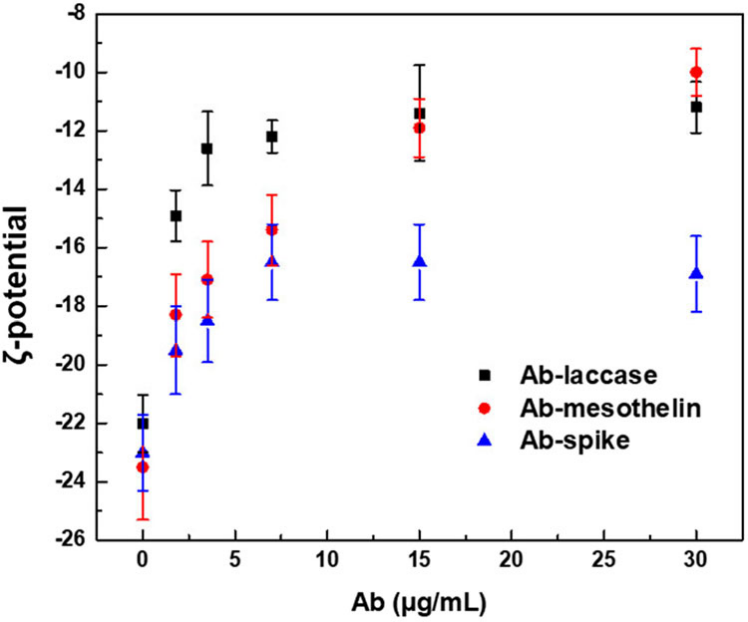
ζ‐potential of Vmh2/SpA‐AuNPs after addition of increasing concentration of Ab‐laccase, Ab‐mesothelin, and Ab‐spike.

Various concentrations of three distinct antibodies—anti‐laccase, anti‐mesothelin, and anti‐spike protein—were tested to verify the efficiency of the system. However, conventional LSPR monitoring was insufficient to confirm complete antibody binding to the AuNPs, as prior functionalization with the protein chimera had already coated the nanoparticle surface, making further detectable peak shifts unlikely. Therefore, ζ‐potential analyses were performed showing changes in surface charge as a function of antibody immobilization and highlighting that concentrations of approximately 15–30 µg/mL, for all tested antibodies, saturate Vmh2/SpA‐AuNPs, as indicated by a plateau in the ζ‐potential values (Figure 2).

**Figure 3:**
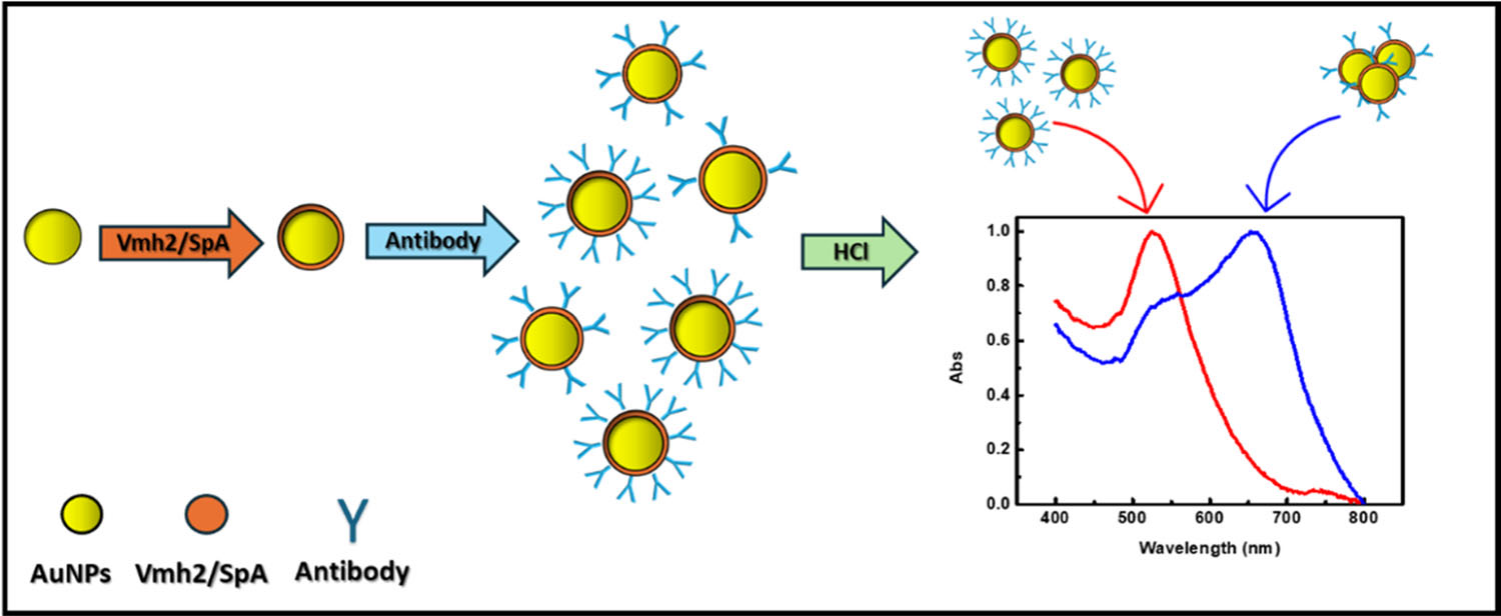
Scheme of the two‐step method.

Furthermore, a salt‐induced aggregation assay—also known as the “two‐step method”—was employed (Anzevino et al. 2024a). This approach leverages the distinct aggregation behaviors of differently antibody‐coated Vmh2/SpA‐AuNPs upon surface charge neutralization due to the addition of HCl as the aggregating agent (see Section 2.7). As a function of the amount of antibody‐coating, Vmh2/SpA‐AuNPs are more stable and less prone to aggregate (Figure 3).

**Figure 4:**
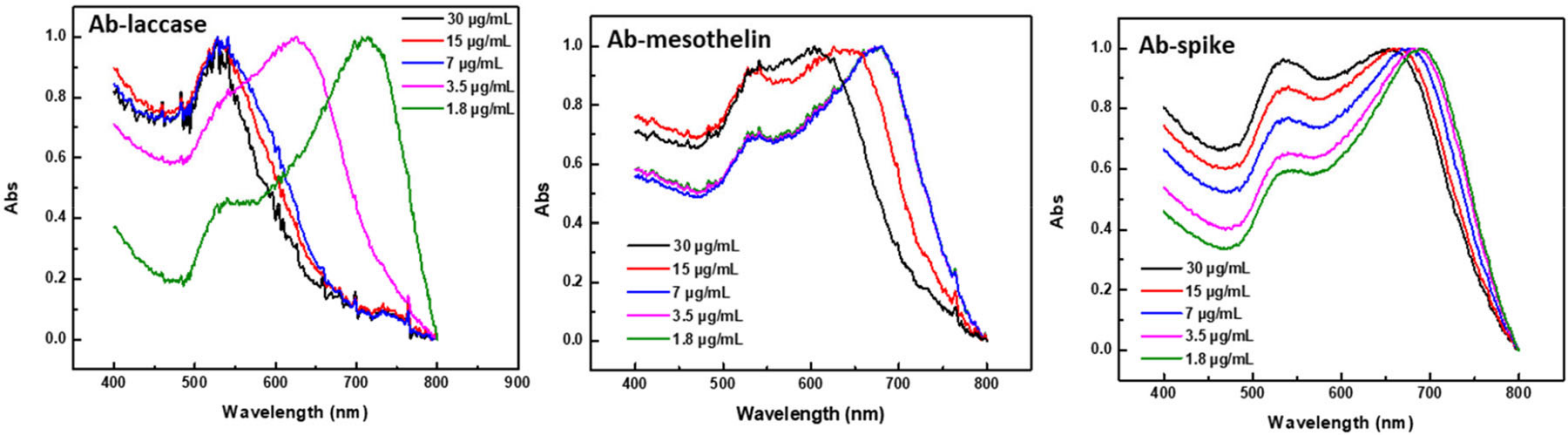
UV‐Vis spectra of Vmh2/SpA‐AuNPs with increasing concentration on Ab‐laccase, Ab mesothelin, and Ab‐spike.

Indeed, as shown in the spectra of Figure 4, Vmh2/SpA‐AuNPs with limited antibody coverage exhibited a pronounced red‐to‐violet color change upon HCl addition, accompanied by broadening of 530 nm peak (monodispersed AuNPs) and the appearance of a high peak in the range 600–800 nm, indicating nanoparticle aggregation. In contrast, spectra of samples incubated with higher antibody concentrations do not change, confirming their colloidal stability due to full surface coverage, as shown also by ζ‐potential analysis.

**Figure 5:**
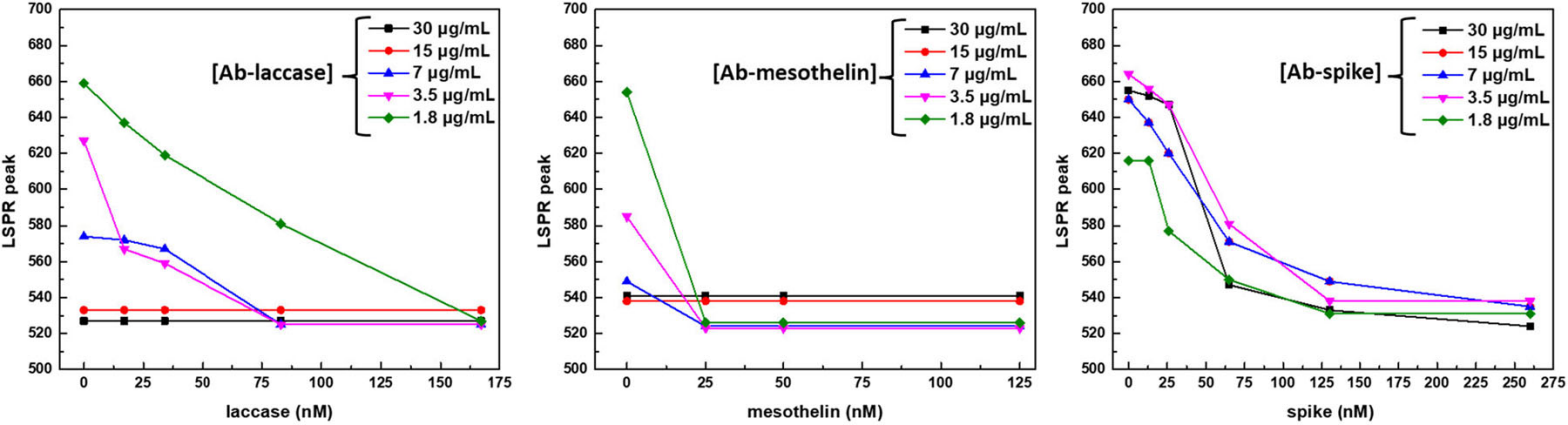
Calibration curves of Vmh2/SpA‐AuNPs incubated with the antibodies anti‐laccase, anti‐mesothelin, and anti‐spike protein at different concentrations (from 1.8 to 30 µg/mL) as a function of their antigen concentrations (17 ÷ 167, 25 ÷ 125, and 13 ÷ 260 nM range for laccase, mesothelin, and spike, respectively).

However, the complete saturation of the AuNPs surface with Vmh2/SpA, stabilizing colloidal suspension, reduces the sensitivity of the biosensors, considering that the analyte‐antibody interaction must be detected through a valuable spectral variation. Therefore, in this system, appropriate antibody concentration must be defined based on the system's responsiveness to the target analyte. To this aim, all Vmh2/SpA‐AuNPs—prepared at varying antibody concentrations—were incubated with increasing amounts of their respective target analytes, and the two‐step method was performed. As shown in Supporting Information S1: Figure S5, at antibody concentration ≤ 7μg/mL, the lower the analyte concentration, the broader the LSPR peak and the higher the peak wavelength shift. An example of the visible variation of the color in this kind of experiment is shown in Supporting Information S1: Figure S6. The calibration curves were built using the wavelength of the LSPR peak (obtained through Gaussian fitting) as the sensing parameter for all pair sets (Figure 5).

This assay was instrumental in identifying the optimal antibody concentration required to achieve the most effective response upon antigen binding. Despite differences in the binding behavior of the three antibodies tested, the data consistently indicated that a concentration of approximately 3.5 µg/mL resulted in the most reliable and reproducible detection performance. Thus, several measurements were performed under the optimal antibody functionalization conditions to construct the three dose–response curves, obtained by the specific recognition of the three different analytes by their respective antibodies.

The LoD achieved for each analyte–antibody pair, as determined by the calibration curves reported in Supporting Information S1: Figure S7, yielded values of 0.38, 0.23, and 550 nM for laccase, mesothelin, and spike proteins, respectively. Notably, the LoD value of 0.23 nM obtained for mesothelin falls within the concentration range relevant for clinical analysis. This sensitivity enables measurements in real samples; in fact, the Vmh2/SpA‐AuNPs remain effective even in tenfold‐diluted serum (Supporting Information S1: Figure S8), a dilution still within the clinically relevant range for mesothelin.

As for the detection of the spike protein, the obtained LoD falls within the nanomolar range, comparable to similar developed colorimetric systems, and could be improved by using an antibody endowed with a higher affinity for its analyte (Singla et al. 2023).

## 4 Conclusion

This study presents the development of a versatile immunosensing platform based on a Vmh2/SpA chimeric protein, combining the adhesive properties of the Class I hydrophobin Vmh2 with the antibody‐binding capabilities of protein A. Vmh2/SpA‐AuNPs demonstrated enhanced antibody immobilization, stability, and biosensing performance. The system was validated using three distinct antibodies, showing sensitive detection through a two‐step method. The Vmh2/SpA‐AuNPs system offers a sustainable, cost‐effective, and flexible solution for immunosensor design, with promising applicability in medical diagnostics, environmental monitoring, and biosensing innovation. Future work may focus on integrating this biointerface into multiplexed or point‐of‐care devices to further expand its utility.

## Author Contributions

**Paola Cicatiello:** conceptualization, methodology, investigation, visualization, writing original draft, funding acquisition. **Bartolomeo Della Ventura:** conceptualization, methodology, visualization, writing original draft. **Giulia Fichera:** methodology, investigation. **Raffaele Velotta:** conceptualization, revision. **Paola Giardina:** conceptualization, writing original draft, supervision. **Alessandra Piscitelli:** conceptualization, writing original draft, supervision, funding acquisition.

## Conflicts of Interest

The authors declare no conflicts of interest.

## Supporting information


**Figure S1:** UV‐Vis spectra of the functionalized nanoparticles exposed to different aggregating agents such as (A) HCl, (B) NaCl, and (C) PBS at various concentrations. **Figure S2:** Western blot analysis of samples at different days recovered by *Pichia pastoris* BG10 growth at the temperature of (A) 28°C and (B) 20°C; Western blotting analysis of Vmh2‐SpA expressed by *P. pastoris* strain BG23 at different days at (C) 28°C without PMSF; (D) 20°C without PMSF; (E) 28°C with PMSF and (F) 20°C with PMSF. **Figure S3:** Western blot analysis of the Vmh2/SpA and the calibration curve with a standard protein. **Figure S4:** UV‐Vis spectra of Vmh2/SpA‐AuNPs‐and SpA‐AuNPs after 4 months. **Figure S5:** UV‐Vis spectra of Vmh2/SpA‐AuNPs incubated with different concentrations of the antibodies anti‐laccase, anti‐mesothelin and anti‐spike protein (from 1.8 to 30 µg/mL) at varying of their analytes concentrations such as 17÷167 nM, 25÷125 nM and 13÷260 nM range for laccase, mesothelin and spike, respectively. **Figure S6:** Color change of the Vmh2/SpA‐AuNPs depending on the antigen concentration. Vmh2/SpA‐AuNPs were functionalized with the antibodies anti‐laccase (from 1.8 to 30 µg/mL) and incubated in multiwell plate with laccase in the range concentrations 17÷167 nM. **Figure S7:** Calibration curves of laccase, mesothelin and spike proteins. **Figure S8**: UV‐Vis spectra of Ab/Vmh2/SpA‐AuNPs in the presence of pure, 10‐ and 100‐ fold diluted serum after HCl addition and nanoparticle aggregation.

## Data Availability

The data that support the findings of this study are available on request from the corresponding author. The data are not publicly available due to privacy or ethical restrictions.
