# Supplementary material for: Gold Nanoparticles Coated With Hydrophobin‐ProteinA Fusion Protein: Development of a Versatile Immunosensing Platform
Source: Biotechnol Bioeng. 2025 Sep 13;122(12):3332–8. doi: 10.1002/bit.70069 (PMC12599489; doi:10.1002/bit.70069)
Supplement: Supplementary file 1 — Figure S1: UV‐Vis spectra of the functionalized nanoparticles exposed to different aggregating agents such as (A) HCl, (B) NaCl, and (C) PBS at various concentrations. Figure S2: Western blot analysis of samples at different days recovered by Pichia pastoris BG10 growth at the temperature of (A) 28°C and (B) 20°C; Western blotting analysis of Vmh2‐SpA expressed by P. pastoris strain BG23 at different days at (C) 28°C without PMSF; (D) 20°C without PMSF; (E) 28°C with PMSF and (F) 20°C with PMSF. Figure S3: Western blot analysis of the Vmh2/SpA and the calibration curve with a standard protein. Figure S4: UV‐Vis spectra of Vmh2/SpA‐AuNPs‐and SpA‐AuNPs after 4 months. Figure S5: UV‐Vis spectra of Vmh2/SpA‐AuNPs incubated with different concentrations of the antibodies anti‐laccase, anti‐mesothelin and anti‐spike protein (from 1.8 to 30 µg/mL) at varying of their analytes concentrations such as 17÷167 nM, 25÷125 nM and 13÷260 nM range for laccase, mesothelin and spike, respectively. Figure S6: Color change of the Vmh2/SpA‐AuNPs depending on the antigen concentration. Vmh2/SpA‐AuNPs were functionalized with the antibodies anti‐laccase (from 1.8 to 30 µg/mL) and incubated in multiwell plate with laccase in the range concentrations 17÷167 nM. Figure S7: Calibration curves of laccase, mesothelin and spike proteins. Figure S8: UV‐Vis spectra of Ab/Vmh2/SpA‐AuNPs in the presence of pure, 10‐ and 100‐ fold diluted serum after HCl addition and nanoparticle aggregation. [file BIT-122-3332-s001.docx]

**Gold nanoparticles coated with Hydrophobin-ProteinA fusion protein: development of a versatile immunosensing platform**

Paola Cicatiello^a^, Bartolomeo Della Ventura^b^, Giulia Fichera^a^, Raffaele Velotta^b^, Paola Giardina^a^, Alessandra Piscitelli^a^


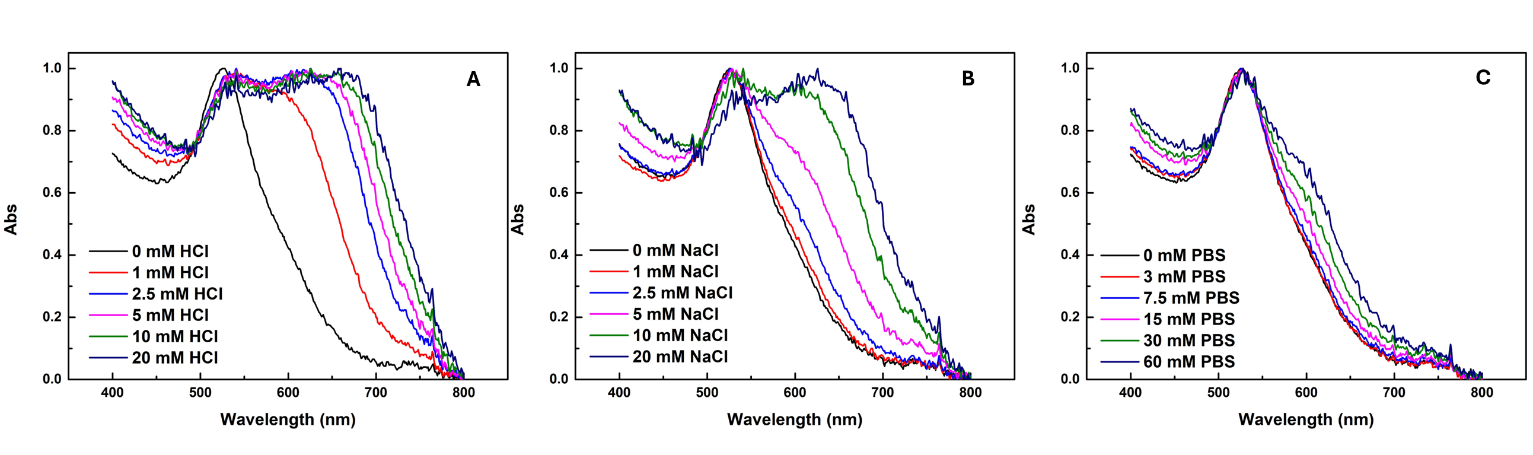


**Figure S1:** UV-Vis spectra of the functionalized nanoparticles exposed to different aggregating agents such as A) HCl, B) NaCl, and C) PBS at various concentrations.


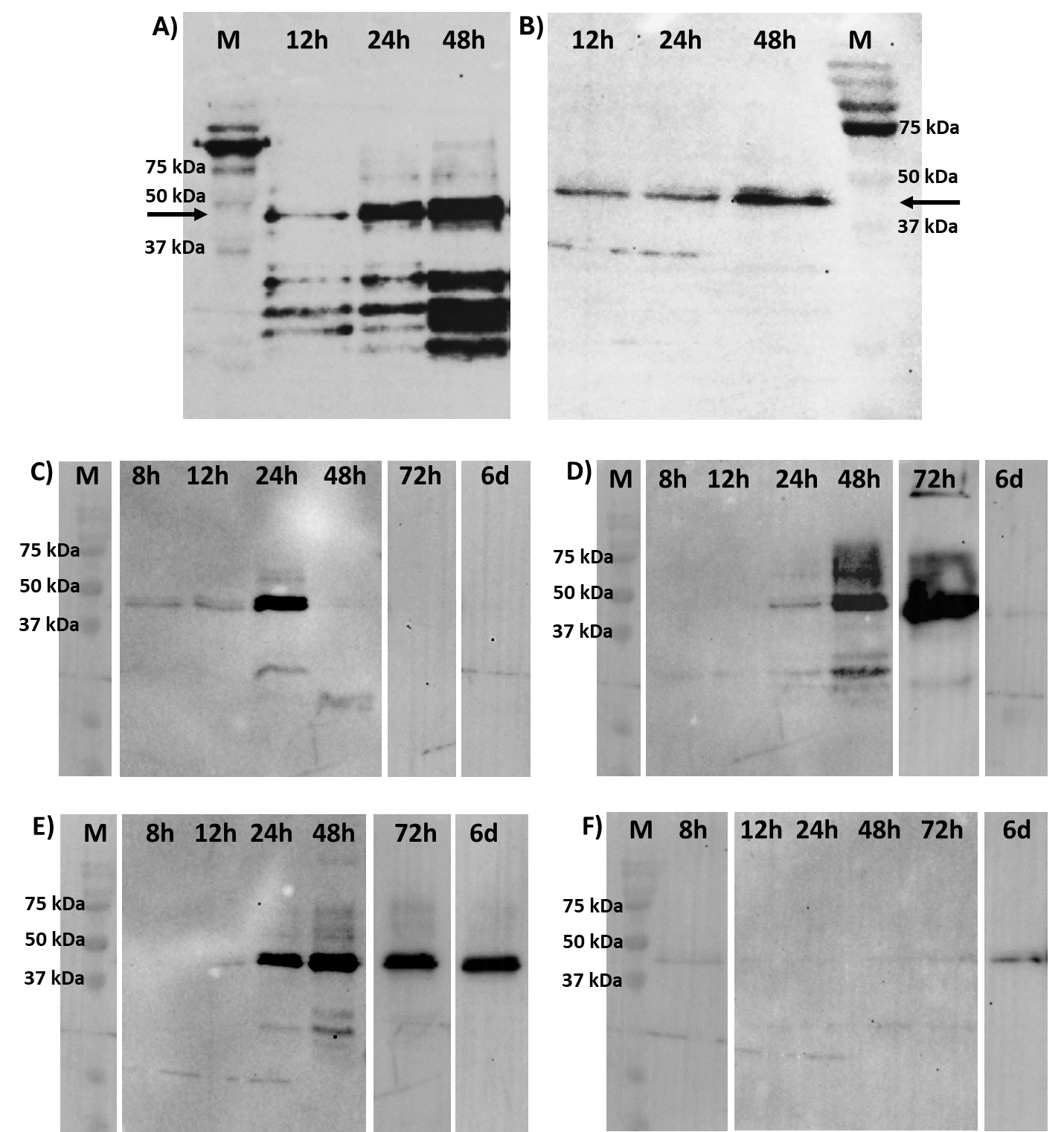


**Figure S2:** Western blot analysis of samples at different days recovered by *Pichia pastoris* BG10 growth at the temperature of A) 28°C and B) 20°C; Western blotting analysis of Vmh2-SpA expressed by *P. pastoris* strain BG23 at different days at C) 28°C without PMSF; D) 20°C without PMSF; E) 28°C with PMSF and F) 20°C with PMSF.


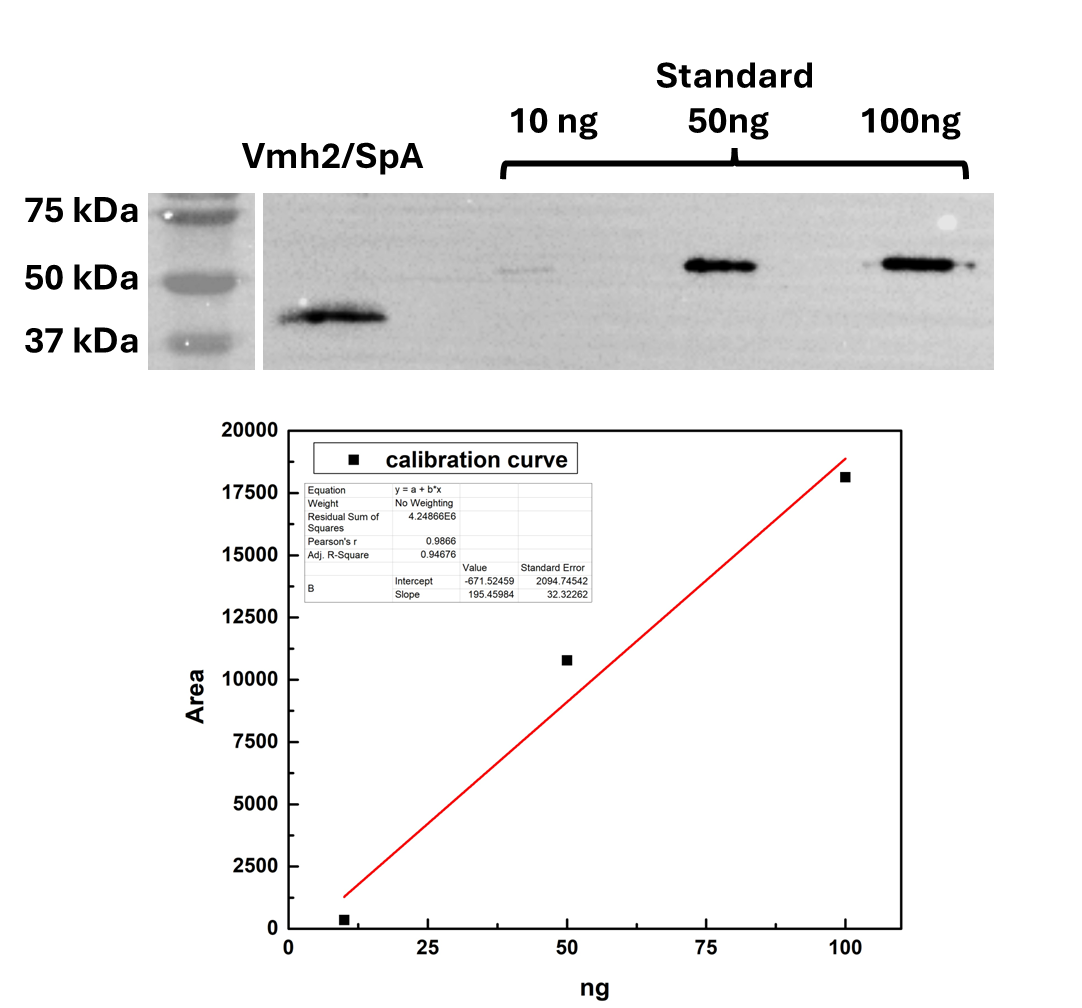


**Figure S3:** Western blot analysis of the Vmh2/SpA and the calibration curve with a standard protein

**Figure S4:** UV-Vis spectra of Vmh2/SpA-AuNPs-and SpA-AuNPs after 4 months.


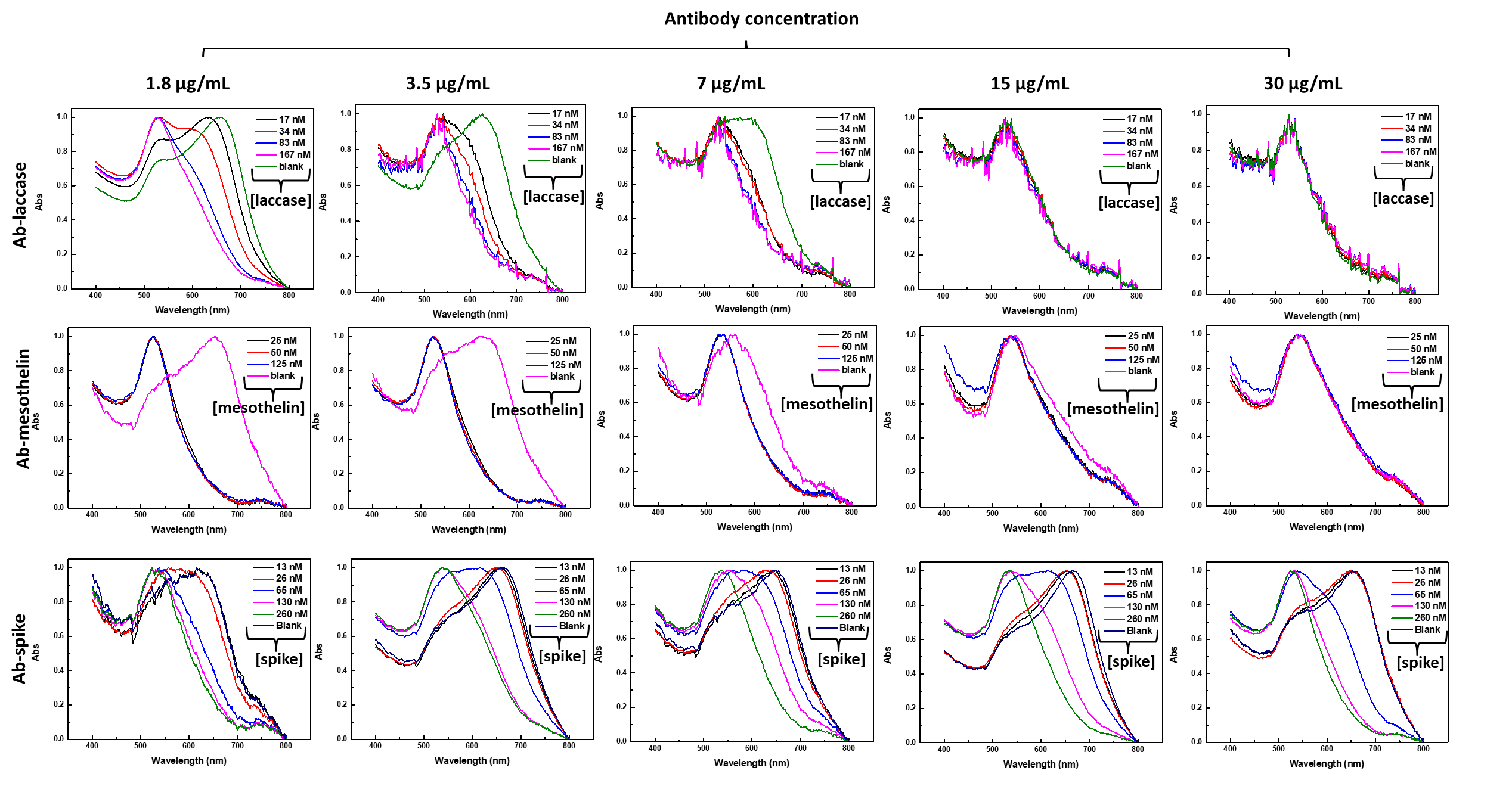


**Figure S5:** UV-Vis spectra of Vmh2/SpA-AuNPs incubated with different concentrations of the antibodies anti-laccase, anti-mesothelin and anti-spike protein (from 1.8 to 30 µg/mL) at varying of their analytes concentrations such as 17÷167 nM, 25÷125 nM and 13÷260 nM range for laccase, mesothelin and spike, respectively.


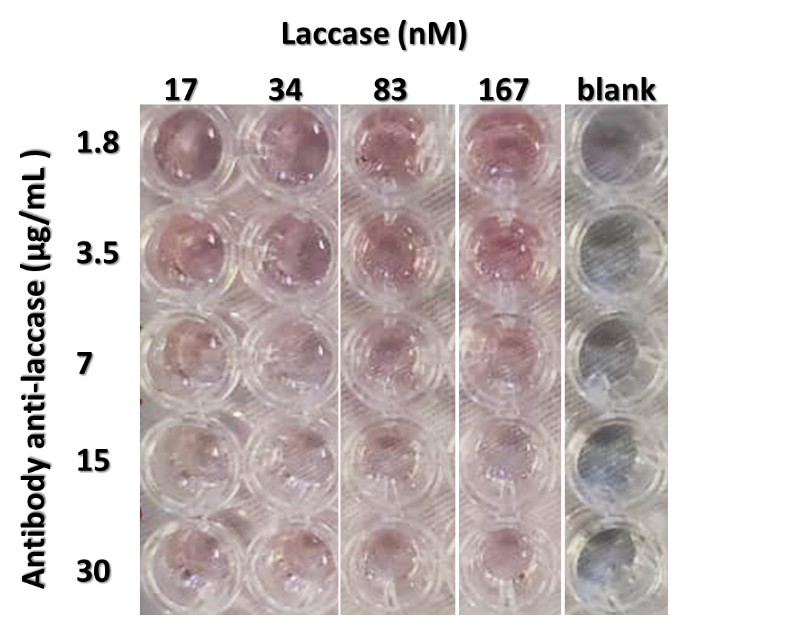


**Figure S6:** Color change of the Vmh2/SpA-AuNPs depending on the antigen concentration. Vmh2/SpA-AuNPs were functionalized with the antibodies anti-laccase (from 1.8 to 30 µg/mL) and incubated in multiwell plate with laccase in the range concentrations 17÷167 nM.


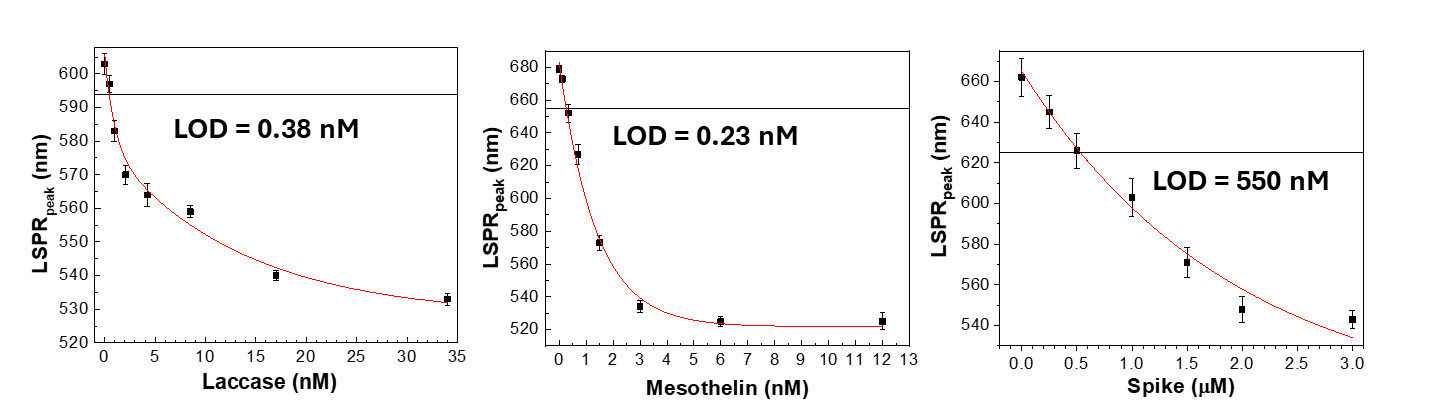


**Figure S7:** Calibration curves of laccase, mesothelin and spike proteins

**Figure S8:** UV-Vis spectra of Ab/Vmh2/SpA-AuNPs in the presence of pure, 10- and 100- fold diluted serum after HCl addition and nanoparticle aggregation.
